# Supplementary material for: Multiple photons enhance quantum machine learning
Source: npj Quantum Inf. 2026 Jun 19;12(1):181. doi: 10.1038/s41534-026-01302-2 (PMC13739719; doi:10.1038/s41534-026-01302-2)
Supplement: Supplementary file 1 [file 41534_2026_1302_MOESM1_ESM.pdf]

SUPPLEMENTARY NOTES

1. Preliminaries 1

2. Quantum Fisher information matrix 2
   2.1. Brief introduction to quantum Fisher information matrix 2
   2.2. Rank of QFIM for Fock states 3

3. Efficient learning of unitaries with Fock states 5
   3.1. Theoretical framework 5
   3.2. Single-photon learning 6
   3.3. Multi-photon learning 6

4. Hardware experiments for learning unitaries 9
   4.1. Brief introduction of SPSA 9
   4.2. Implementation details 9

5. Quantum metric learning with multi-photon states 11
   5.1. Theoretical framework 11
   5.2. Experimental implementation details 11
   5.3. Numerical simulations with larger photon numbers 14

Supplementary References 15

1. PRELIMINARIES

We consider linear optical systems with Fock states, i.e., states with fixed photon numbers. Our terminology follows the conventions developed in boson sampling [1]. The Hilbert space of a system is spanned by Fock states of the form

$$|n_1, n_2, \cdots, n_m\rangle = \frac{1}{\sqrt{n_1! n_2! ... n_m!}} (\hat{a}_1^\dagger)^{n_1} (\hat{a}_2^\dagger)^{n_2} \cdots (\hat{a}_m^\dagger)^{n_m} |\text{vac}\rangle \ , \tag{1}$$

where $\hat{a}_i^\dagger$ is the creation operator of the ith mode, n_i is the photon number in the ith mode, m is the number of modes, and $|\text{vac}\rangle$ is the vacuum state. The system evolution is described by an $m \times m$ unitary matrix U parameterized by K tunable real parameters $\boldsymbol{\theta} = \{\theta_s\}_{s=1}^K$ as

$$U(\boldsymbol{\theta}) = \prod_{s=1}^{K} u_s(\theta_s). \tag{2}$$

In linear optics, the parameters are typically realized as phase shifts within Mach-Zehnder interferometers (MZIs). Note that the parameterization of U is not unique, and there exist many different equivalent configurations. The minimum number of real parameters to realize arbitrary unitary transformations is $m^2 - 1$, which can be efficiently implemented using MZI-based architectures [2, 3].

For an input Fock state $|\phi\rangle$ and unitary matrix U, the probability of measuring a specific output Fock state $|\psi\rangle$ is determined by the permanent of a matrix A, which is constructed from the input state $|\phi\rangle = |n_1, n_2, \cdots, n_m\rangle$, the measured output state $|\psi\rangle = |n'_1, n'_2, \cdots, n'_m\rangle$ and U[1]

$$\langle\psi| \tilde{U} |\phi\rangle = \frac{\text{perm}(A)}{\prod_i n_i! \prod_i n'_i!}. \tag{3}$$

Here, $\tilde{U}$ is a $d \times d$ unitary matrix determined by U, where $d = \binom{n+m-1}{n}$ is the dimension of the Fock space, corresponding to the number of photon occupation configurations for n photons in m modes. For pure input states, the evolution can also be described in the Schrödinger picture using the density matrix formalism $\tilde{\rho} = \tilde{U}\rho\tilde{U}^\dagger$, where ρ and $\tilde{\rho}$ denote the density matrices of the input and output states, respectively.

2. QUANTUM FISHER INFORMATION MATRIX

2.1. Brief introduction to quantum Fisher information matrix

What is the capability of a trainable parameterized quantum circuit for quantum machine learning (QML) tasks? This question has been explored through the concept of quantum effective dimension [4], which describes the ability of a given model to express quantum states. While previous studies have primarily focused on qubits, here we apply these measures to linear optical circuits with Fock states.

Consider a parameterized quantum state $U(\boldsymbol{\theta})\,|\phi\rangle \equiv |\psi(\boldsymbol{\theta})\rangle$, which is generated by applying a parameterized unitary transformation $U(\boldsymbol{\theta})$ to a fixed input state $|\phi\rangle$. The expressive power or capacity of such states can be characterized using the quantum Fisher information matrix (QFIM) [4]. For pure states the QFIM is defined as [5]

$$\mathcal{F}_{ij} = 4\mathrm{Re}(\langle\partial_i\psi|\partial_j\psi\rangle - \langle\partial_i\psi|\psi\rangle\,\langle\psi|\partial_j\psi\rangle)\,, \tag{4}$$

where $|\partial_i\psi\rangle$ denotes the derivative of $|\psi\rangle$ with respect to the ith component of $\boldsymbol{\theta}$. The QFIM can be interpreted as a metric that quantifies how a small perturbation in $\boldsymbol{\theta}$ affects the resulting quantum state as follows

$$|\,\langle\psi(\boldsymbol{\theta})|\psi(\boldsymbol{\theta}+\mathrm{d}\boldsymbol{\theta})\rangle\,|^2 = 1 - \frac{1}{4}\mathcal{F}_{ij}\mathrm{d}\boldsymbol{\theta}^i\mathrm{d}\boldsymbol{\theta}^j\,. \tag{5}$$

The quantum effective dimension $D_1 = \mathrm{rank}(\mathcal{F})$ is given by the rank of the QFIM, which captures the number of independent directions in the state space that can be explored by $|\psi(\boldsymbol{\theta})\rangle$ [4]. When the rank reaches its maximum and no longer increases with the addition of more circuit parameters, the circuit is said to be overparameterized, indicating that it is capable of exploring all accessible directions.

While the QFIM characterizes the capacity of a single parameterized state, QML tasks typically involve a set of training states. To properly describe such scenarios, one can generalize the QFIM to the data quantum Fisher information matrix (DQFIM), which describes the space of $U(\boldsymbol{\theta})$ that can be learned using multiple training states [6]. Specifically, consider a parameterized unitary $U(\boldsymbol{\theta}) \equiv U$ acting on a set of L training states $T_L = \{|\psi_\ell\rangle\}_{\ell=1}^L$, i.e., $U(\boldsymbol{\theta})\,|\psi_\ell\rangle$. We define the data state as

$$\rho_L = \frac{1}{L}\sum_{\ell=1}^{L}|\psi_\ell\rangle\,\langle\psi_\ell|\,, \tag{6}$$

which is the mixture of all training states. The data quantum Fisher information metric (DQFIM) is given by [6]

$$\mathcal{Q}_{ij}(\rho_L) = 4\mathrm{Re}(\mathrm{tr}(\partial_i U\rho_L\partial_j U^\dagger) - \mathrm{tr}(\partial_i U\rho_L U^\dagger)\mathrm{tr}(U\rho_L\partial_j U^\dagger))\,. \tag{7}$$

The rank of the DQFIM is given by

$$D_L(n) = \mathrm{rank}(\mathcal{Q})\,, \tag{8}$$

which denotes the number of learnable independent parameters of U that can be learned using dataset T_L with a circuit of K parameters. Increasing the number of circuit parameters K enhances the expressiveness of the model, however only up to a limit. The maximal rank of the DQFIM, where one maximizes over the number of circuit parameters K,

$$R_L(n) = \max_K \mathrm{rank}(\mathcal{Q}) \tag{9}$$

measures the maximal possible number of independent parameters of U that can be learned via the training set T_L.

Now, we ask what is the maximal effective dimension for linear optical circuits. Consider a system of n indistinguishable photons distributed over m modes. Naively, the full Hilbert space can be written as $\mathbb{C}^{(n+1)^m}$, assuming each mode can host up to n photons. However, a fixed total number of photons n reduces the accessible Hilbert space to Fock basis states of the form $|n_1, n_2, \ldots, n_m\rangle = |n_1\rangle\,|n_2\rangle \ldots |n_m\rangle$ with $\sum_{i=1}^m n_i = n$ and $n_i \geq 0$. A simple combinatorial argument shows that the number of possible basis states is $\binom{n+m-1}{n}$, corresponding to the number of ways to choosing $m-1$ dividers to split n into m non-negative integers. Alternatively, in first quantization, the state of the system can be expressed as a tensor product of n photons $\otimes_{b=1}^n |\psi_b\rangle$ with wavefunction $|\psi_b\rangle$ of the bth photon. To construct the full many-body wavefunction, one has to take all symmetric combinations of the state.

However, since we apply only a linear optical unitary U acting on m modes, rather than a general unitary over the full Hilbert space, the set of accessible states is confined to a polynomially sized subspace within the exponentially

large Hilbert space. While a straightforward parameterization of U appears to have $2m^2$ real parameters, the actual number of independent parameters R_∞ is lower due to constraints on U. First, the unitarity condition $\mathrm{tr}(UU^\dagger) = I$ imposes m^2 constraints. Second, the unitary is invariant under global phase rotations $U \to e^{i\phi}U$, introducing one additional constraint. Together, these reduce the number of independent real parameters to $R_\infty(n) = m^2 - 1$, which corresponds to the maximum number of degrees of freedom for a linear optical unitary acting on m modes, regardless of the photon number n. For linear optics, U acts on each photon individually, so it is sufficient to consider the transformation of individual photons when analyzing the system evolution.

2.2. Rank of QFIM for Fock states

We now want to understand the QFIM for a parameterized linear optical unitary U applied to a single Fock state, i.e., one training state with $L = 1$. A linear optical unitary can be written as an $m \times m$ dimensional matrix with entries u_{ks} written in the basis of optical modes $\{|s\rangle\}_{s=1}^m$

$$U = \sum_{k,s=1}^{m} u_{ks} |k\rangle \langle s| \,, \tag{10}$$

and is applied to a Fock state $|n_1, n_2, \dots, n_m\rangle$. First, we consider the simplest case $n = 1$ with state $|n_1 = 1\rangle$, where only mode $s = 1$ is occupied and all other modes are empty. Without loss of generality, we assume that the photon is initially in the first mode. In this case, we have $|\psi_1(U)\rangle = U|n_1 = 1\rangle = \sum_{k=1}^m u_{k1} |n_k = 1\rangle$. Note that our approach is general for any single-photon state, as arbitrary state $\sum_k \alpha_k |n_k = 1\rangle$ can be transformed into this form via some fixed unitary V such that $|n_1 = 1\rangle = V \sum_k \alpha_k |n_k = 1\rangle$, and the total transformation becomes $U' = UV^\dagger$.

How many independent parameters of U can we learn by performing measurements on the state $|\psi(U)\rangle$? This can be solved through a simple counting argument [7]. The state

$$|\psi_1(U)\rangle = \sum_{k=1}^{m} u_{k1} |n_k = 1\rangle \tag{11}$$

depends only on the first column vector of U. Let us write $U = (u_1, \dots, u_m)$, where $u_1 = (u_{11}, u_{21}, \dots, u_{m1})^{\mathrm{T}}$ is the first column vector. By performing measurements on $|\psi_1\rangle$ and varying the parameters, one can learn about the column vector u_1. It has $2m$ real parameters as we can parameterize its coefficients as $u_{k1} = a_{k1} + ib_{k1}$. However, the parameters that can be learned are actually fewer because of the constraints on the state. In particular, the normalization condition $\langle\psi_1|\psi_1\rangle = 1$ implies $u_1^\dagger u_1 = 1$ and reduces the learnable parameters by one. Additionally, we have invariance under phase rotations, i.e., $u_1 \to e^{i\phi}u_1$ is not observable. Thus, from $|\psi_1\rangle$ we can learn

$$R_1(n = 1) = 2m - 2 \tag{12}$$

parameters of the column vector u_1.

Similarly, coherent states (such as those produced by lasers) without squeezing transformed by linear optical circuits are subject to the same bound as single photons, i.e.,

$$R_1^{\text{coherent}} = R_1(n = 1) = 2m - 2\,, \tag{13}$$

as we now proceed to show. Coherent states in m modes are written as [8]

$$|\psi_{\mathrm{c}}(\alpha)\rangle = \exp\!\left(-|\alpha|^2/2\right) \exp\!\left(\sum_{i=1}^{m} \alpha_i \hat{a}_i^\dagger\right) |\mathrm{vac}\rangle \,, \tag{14}$$

where $|\mathrm{vac}\rangle$ is the vacuum state, $\alpha_i \in \mathbb{C}$ are the complex amplitudes, and $\hat{a}_i^\dagger$ is the photon creation operator for mode i. A linear optical unitary U simply transforms the creation and annihilation operators as $U\hat{a}_i^\dagger \to \sum_k u_{ki}^* \hat{a}_k^\dagger$. Without loss of generality, let us assume that only the first mode of the input coherent state is occupied, i.e., $e^{-|\alpha|^2/2} e^{\alpha \hat{a}_1^\dagger} |\mathrm{vac}\rangle$. Applying the unitary transformation U yields

$$|\psi_{\mathrm{c}}(\alpha)\rangle = \exp\!\left(-|\alpha|^2/2\right) \exp\!\left(\alpha \sum_{k=1}^{m} u_{k1}^* \hat{a}_k^\dagger\right) |\mathrm{vac}\rangle \,. \tag{15}$$

Thus, the transformed state depends only on the first column of U, and the number of learnable parameters is the same as in the single-photon case.

Next, we consider the case of $n = 2$ where the photons are in two different modes, $n_1 = 1$ and $n_2 = 1$. From the photon in mode $s = 1$, we learn $2m - 2$ independent parameters of the column vector u_1. The second photon, occupying mode $s = 2$, reveals the state (written in first quantization)

$$|\psi_2\rangle = \sum_{k=1}^{m} u_{k2} |k\rangle \ , \tag{16}$$

which depends on the second column vector u_2 of U, also containing $2m$ real parameters. Due to normalization, we loose one parameter from the constraint $u_2^\dagger u_2 = 1$. Further, the overall phase of u_2 is unobservable, eliminating another parameter. Finally, unitarity requires that the first and second column vectors to be orthogonal, which implies $u_1^\dagger u_2 = 0$. This removes two parameters as the real and complex parts must be both zero. Thus, the number of additional learnable parameters from u_2 is $R_1(n = 2) - R_1(n = 1) = 2m - 4$. Combining u_1 and u_2, we get

$$R_1(n = 2) = 4m - 6 \tag{17}$$

free parameters that can be learned using two photons in orthogonal modes.

We now consider the incremental gain in learnable parameters when increasing the photon number from n to $n+1$. Assume we begin with the Fock state $|F_n\rangle = |11 \dots 1100 \dots 0\rangle$, where n photons occupy the first n modes. This allows us to learn $R_1(n)$ independent real parameters from the corresponding column vectors $u_1, \dots, u_n$ of the unitary U. If we include an additional photon in the $(n+1)$th mode, the output state for this photon is $|\psi_{n+1}\rangle = \sum_{k=1}^{m} u_{k,(n+1)} |k\rangle$, which depends on the column vector u_{n+1}. Similarly, this column vector contains $2m$ parameters, of which two are removed due to normalization and invariance under overall phase. The new vector u_{n+1} must be orthogonal to all previous column vectors $u_1, \dots, u_n$, yielding $2n$ constraints. In total, we can learn

$$\Delta_n = R_1(n + 1) - R_1(n) = 2m - 2n - 2 \tag{18}$$

additional independent parameters.

Let us now sum the incremental gains and get the explicit expression for $R_1(n)$

$$R_1(n) = \sum_{k=0}^{n-1} \Delta_k = n(2m - n - 1)\,. \tag{19}$$

The growth of $R_1(n)$ stops when $\Delta_{m-1} = 0$, which occurs at $n = m-1$. This means that with $n = m-1$ photons, the QFIM reaches its maximum rank, and no additional learnable parameters can be obtained by adding more photons. For $n \geq m - 1$, the number of learnable parameters using Fock states satuates at $R_1(m - 1) = m^2 - m$. Hence, we can express the QFIM rank $R_1(n, m)$ as

$$\begin{aligned} R_1(n, m) &\leq 2mn - n^2 - n \quad (n \leq m - 1)\,, \\ R_1(n, m) &= m^2 - m \qquad\quad\ \ (n \geq m - 1)\,. \end{aligned} \tag{20}$$

This result shows that the number of learnable independent parameters for a single Fock state grows linearly with the number of modes m, but only sub-linearly with the number of photons n.

Finally, we consider the general case of multiple training states $L > 1$ to compute $R_L(n)$. Here, we count the degrees of freedom of U that can be learned by using a dataset of L training states $\{|\psi_\ell\rangle\}_{\ell=1}^{L}$. Each $|\psi_\ell\rangle$ is an arbitrary n-photon Fock state, and is able to reveal at most n columns of U. Thus, across the full dataset, up to nL columns can be accessed. One can now compute R_L as the number of independent parameters of nL columns of the $m \times m$ unitary U. This has been derived in Ref. [6], where the (tight) upper bond is given by

$$\begin{aligned} R_L(n, m) &\leq 2mnL - n^2L^2 - 1 - \delta_{L,1}(n - 1) \qquad (nL \leq m)\,, \\ R_L(n, m) &= m^2 - 1 - \delta_{L,1}(m - 1) \qquad\qquad\quad (nL > m)\,. \end{aligned} \tag{21}$$

Notably, the case $L = 1$ as derived in (20) is a special case, where the number of parameters is reduced due to the fact that a single Fock state cannot learn any local phases. In contrast, $L > 1$ matches Ref. [6].

Eq. (20) is verified in Fig. 2c in the main text for the case $m = 6$ and $n = 1, 2, 3, 4, 5$. Here, we further verify the general expression in Eq. (21) through numerical simulations with $m = 6$, $n = 2$ and varying numbers of L. The results are presented in Fig. S1. As the number of trainable parameters increases, the circuit becomes overparameterized and the DQFIM rank saturates at 18, 31, and 35 for different values of L, which are in agreement with the values given by (21).

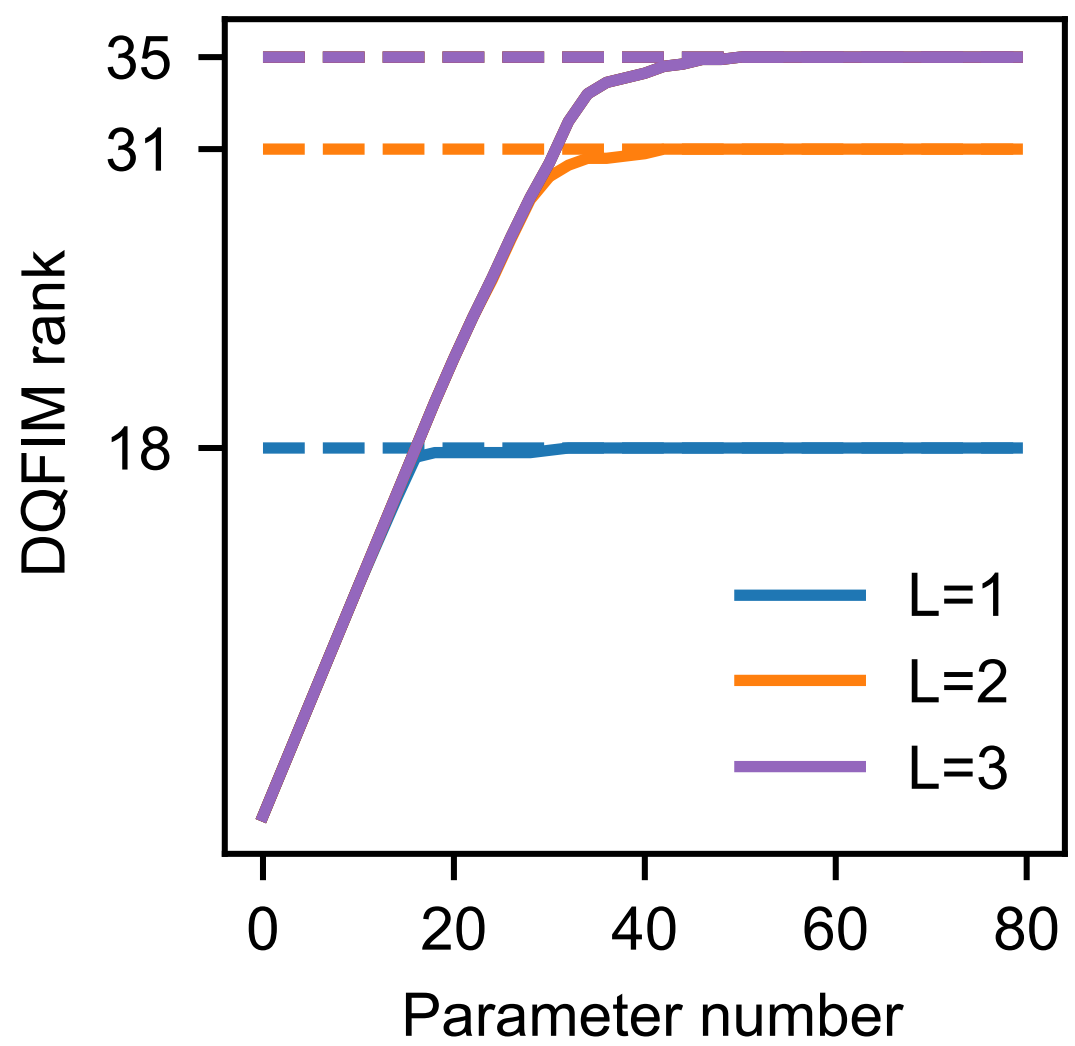

Supplementary Figure S1. **Simulation of the DQFIM rank D_L with multiple training states.** D_L is shown against number of circuit parameters K for different numbers of training states $L = 1, 2, 3$ and a linear optical circuit with $m = 6$ modes and $n = 2$ photons.

3. EFFICIENT LEARNING OF UNITARIES WITH FOCK STATES

3.1. Theoretical framework

We now study the problem of learning an unknown unitary V of a linear optical system. We assume that we only have oracle access to V: the internal structure of V is unknown, and we are only allowed to apply V to a set of training states of fixed dimension. The goal is to learn V by applying it to these training states, performing measurements, and inferring from the measurement outcomes.

To achieve this, we optimize a parameterized linear optical unitary $U(\boldsymbol{\theta})$, and we aim to find optimal parameters $\boldsymbol{\theta}^*$ such that

$$U(\boldsymbol{\theta}^*) = V \tag{22}$$

up to an irrelevant global phase. In general, to faithfully represent an m-mode unitary V, $U(\boldsymbol{\theta})$ must have at least $m^2 - 1$ real parameters. In this case, the model is said to be overparameterized. Overparameterized models are capable of fully exploring the underlying quantum state space, and as a result, they usually converge to the global minimum during training [6, 9, 10].

To train the model, we use a dataset of L input quantum states defined as

$$T_L^n = \{|\psi_\ell\rangle = W_\ell\,|\bar{n}\rangle\}_{\ell=1}^{L}\,, \tag{23}$$

where W_ℓ is a unitary for preparing the training state $|\psi_\ell\rangle$, and $|\bar{n}\rangle$ is a Fock state of n photons with at most one photon per mode. The learning process optimizes the parameters $\boldsymbol{\theta}$ such that

$$U(\boldsymbol{\theta})\,|\psi_\ell\rangle = V\,|\psi_\ell\rangle\,,\ \forall\ell = 1,\dots,L\,. \tag{24}$$

In practice, this is achieved by maximizing the cost function $C = L^{-1}\sum_{\ell=1}^{L} C_\ell(\boldsymbol{\theta})$ with

$$C_\ell(\boldsymbol{\theta}) = |\,\langle\psi_\ell|\,V^\dagger U(\boldsymbol{\theta})\,|\psi_\ell\rangle\,|^2 = |\,\langle\bar{n}|\,W_\ell^\dagger V^\dagger U(\boldsymbol{\theta}) W_\ell\,|\bar{n}\rangle\,|^2 \tag{25}$$

for all ℓ.

We now ask: what is the minimal number of training states $L_{\mathrm{c}}(n, m)$ required to fully reconstruct an unknown unitary V as a function of the photon number n of the states in the training set and the number of modes m of V? We show that this is possible when the DQFIM rank reaches its maximal value $R_L = m^2 - 1$, leading to the bound

$$L_{\mathrm{c}}(n, m) = \mathrm{ceil}\left(\frac{m-1}{n}\right) + 1\,, \tag{26}$$

where ceil(x) denotes the smallest integer greater than or equal to x. In particular, for $n = 1$ we have $L_\mathrm{c} = m$, while for $n \geq m - 1$ we have $L_\mathrm{c} = 2$ only.

Additionally, we find similar bounds for a slightly relaxed problem as studied in the main text, where we learn V only up to local phase rotations and mode permutations. That is, the learned unitary may differ from V by a diagonal phase unitary $F(\boldsymbol{\phi}) = \sum_{j=1}^{m} e^{-i\phi_j} |j\rangle\langle j|$ as well as a permutation operator P_σ which permute modes, e.g., $P_\sigma |10\rangle = |01\rangle$. Generally for quantum interference experiments, a unitary of this kind could be applied (as multiplication) to the right or to the left of unknown unitary, without altering the experiment. In this case, the number of required training states is

$$L_\mathrm{c}^\mathrm{p}(n, m) = \mathrm{ceil}\left(\frac{m-1}{n}\right) , \tag{27}$$

and the maximal DQFIM rank is lower, given by $R_L = m^2 - m$.

This demonstrates a fundamental advantage in QML in terms of the number of training states required for learning. Specifically, single-photon states (as well as coherent light such as laser fields) require a number of training states $L \propto m$ which scales with the number of modes m. In contrast, Fock states with $n \propto m$ require only a constant number of training states $L = \mathrm{const}$.

3.2. Single-photon learning

We start by describing the training algorithm for the case $n = 1$, i.e., training with single-photon Fock states. Note that the results also apply to coherent states such as laser light, which behave similarly under linear optical transformations.

For the training set T_L^1, we select L state preparation unitaries W_ℓ, each applied on a single-photon state $|\bar{n} = 1\rangle$. To learn the target unitary V, one maximizes the cost function

$$C(\boldsymbol{\theta}) = \frac{1}{L}\sum_{\ell=1}^{L} C_\ell(\boldsymbol{\theta}) . \tag{28}$$

As shown in Ref. [6], to fully learn a general m-dimensional unitary, one requires at least $L = m$ training states. This result follows from the rank of the DQFIM, which reaches its maximum only when $L \geq m$. The reason is intuitive: to fully reconstruct V, we must be able to learn each of its coefficients v_{ij}. Let us consider the training state $|10\ldots0\rangle$, i.e., a single photon in the first mode. After applying V, one gets

$$V|10\ldots0\rangle = \sum_{i=1}^{m} v_{i1}|i\rangle , \tag{29}$$

where $|i\rangle$ denotes a single photon in the ith mode. Thus, this state provides information only about the first column of V. To fully learn V, we need at least $L \geq m$ linear independent training states to learn all columns. If we choose $L < m$, not all columns of V are revealed, implying that we cannot completely learn V.

Note that if we want to learn V only up to local phases, $m - 1$ training states are sufficient. This is because the final column of V does not need to be uniquely determined in this case.

3.3. Multi-photon learning

We now extend our analysis to learning with Fock states containing n photons. By the same reasoning as in the single-photon case, each training state now provides access to n columns of V. Consequently, fewer training states are required. For example, consider a training state $|110\ldots0\rangle$ with $n = 2$ photons. This state can be expressed in terms of bosonic creation operators as $|110\ldots0\rangle = a_1^\dagger a_2^\dagger|\mathrm{vac}\rangle$, where $a_j^\dagger$ creates a photon in mode j, and $|\mathrm{vac}\rangle$ denotes the vacuum state. Under the action of a linear optical unitary V, the creation operators transform as $Va_j^\dagger = \sum_{j=1}^{m} v_{ij}a_i^\dagger$ [11]. Applying V yields

$$V|110\ldots0\rangle = (\sum_{i=1}^{m} v_{i1}a_i^\dagger)(\sum_{j=1}^{m} v_{j2}a_j^\dagger)|\mathrm{vac}\rangle . \tag{30}$$

In particular, we see that the transformed state now depends on the first and second columns of V, allowing one to learn both of them simultaneously.

However, there are two issues to consider when learning with multi-photon Fock states. Firstly, Fock states do not reveal information about local phase rotations. Consider the unitary consisting of mode-wise phase shifts $F(\boldsymbol{\phi}) = \sum_{j=1}^{m} e^{-i\phi_j} |j\rangle\langle j|$ with phase ϕ_j on the jth mode. This unitary leaves any Fock state invariant, e.g., for $n = m = 4$

$$F(\boldsymbol{\phi})\,|1111\rangle = |1111\rangle\ . \tag{31}$$

As a result, to learn local phases, one requires a training state that is not a Fock state, but a coherent superposition of different modes such that $F(\boldsymbol{\phi})$ acts non-trivially on the state.

Secondly, consider learning a unitary that implements a permutation operation P_σ, which permute modes according to some permutation function $\sigma(j) = k$, with $j, k \in \{1, 2, \ldots, m\}$. For example, for $m = 3$, the permutation function with $\sigma(1) = 3$, $\sigma(2) = 1$, and $\sigma(3) = 2$ is a cyclic permutation of modes $(1, 2, 3) \to (3, 1, 2)$.

Crucially, permutations that only affect modes occupied by photons leave Fock states invariant. For example, consider P_σ that swaps the first and second modes $\sigma(1) = 2$ and $\sigma(2) = 1$. Applying this to the Fock state $|1100\rangle$, we find $P_\sigma\,|1100\rangle = |1100\rangle$, indicating that the state is unchanged. As the unitary P_σ has no effect on the Fock state, it cannot be distinguished from the identity operation, and thus cannot be correctly learned. This implies that when training with multi-photon Fock states, the learned unitary $U(\boldsymbol{\theta})$ can learn the target unitary V only up to a permutation of the occupied modes. In practice, we observe that these permutation symmetries cause training to converge to local minima, even when $U(\boldsymbol{\theta})$ is overparameterized. That is, the model may become trapped in a solution associated with the wrong permutation symmetry. As a consequence, using the same training protocol as in the single-photon case becomes ineffective for the multi-photon regime.

To mitigate these problems, we now propose an efficient learning algorithm for m-mode unitaries using n-photon Fock states, which unifies the single-photon and multi-photon cases within a common framework. We represent Fock states as $|\boldsymbol{s}\rangle = |s_1, s_2, \ldots, s_m\rangle$, where s_j is the number of photons in the jth mode and $\sum_j s_j = n$. This protocol requires a total of $L(n, m) = \text{ceil}\left(\frac{m-1}{n}\right) + 1$ training states, consisting of $L - 1$ multi-photon Fock states with $n \geq 1$ and one single-photon state. It proceeds in three steps:

1. Train the parameterized unitary $U(\boldsymbol{\theta})$ using a training set consisting of $L - 1$ n-photon Fock states as $T_{L-1} = \{|\boldsymbol{s}^{(\ell)}\rangle\}_{\ell=1}^{L-1}$. To ensure that V can be learned correctly (up to local phases and mode permutations), we require that for each mode $j = 1, 2, \ldots, m-1$, there must be at least one photon across the training set, i.e., $\sum_{\ell=1}^{L-1} s_j^{(\ell)} > 0$. We then minimize the loss function

$$C(\boldsymbol{\theta}) = 1 - \frac{1}{L} \sum_{\ell=1}^{L-1} |\langle \boldsymbol{s}^{(\ell)} | V^\dagger U(\boldsymbol{\theta}) | \boldsymbol{s}^{(\ell)} \rangle|^2\,. \tag{32}$$

   For $L-1 \geq \text{ceil}(\frac{m-1}{n})$, the union of the training states occupies at least $m-1$ modes. This condition is sufficient to saturate the rank of the DQFIM at $m^2 - m$, which is the number of independent parameters of an $m \times m$ unitary up to local phases. If the learning task only requires identifying V up to local phase shifts and mode permutations, the algorithm can stop here. In that case, the minimum number of required training states is $L_{\text{c}}^{\text{p}}(n, m) = \text{ceil}\left(\frac{m-1}{n}\right)$.

   In the next two steps, an additional single-photon training state is used to learn the local phase and mode permutation, completing the full learning of V. With this additional state, the DQFIM reaches its theoretical maximum rank of $m^2 - 1$, implying that all independent parameters of the unknown unitary V can be learned.

2. We now describe how to resolve the mode permutation ambiguity. Notably, this step does not require variational learning and can be performed efficiently by measuring a single-photon state $|\psi_L\rangle = W\,|\bar{n} = 1\rangle$, with W drawn from the Haar measure. We measure the occupation probabilities across all output modes for both the trained unitary $U(\boldsymbol{\theta}^*)$ and the target unitary V. These are defined as

$$v_j = |\,\langle j|\, U(\boldsymbol{\theta}^*) W\,|\bar{n} = 1\rangle\,|^2\,, v'_j = |\,\langle j|\, V W\,|\bar{n} = 1\rangle\,|^2\,. \tag{33}$$

   By comparing the probabilities, we can obtain the permutation P_σ (a bijective function which maps each mode $j \to j'$) such that distributions v_j and v'_j match, i.e., $v'_j = P_\sigma v_j$ for all $j = 1, \ldots, m$. P_σ can be implemented with linear optical circuits as a sequence of beam splitters which swap modes.

3. Finally, we determine the correct local phases $\boldsymbol{\phi}$ associated with a diagonal phase unitary

$$F(\boldsymbol{\phi}) = \sum_{j=1}^{m} e^{-i\phi_j} \left|j\right\rangle \left\langle j\right| . \tag{34}$$

$F(\boldsymbol{\phi})$ is optimized with the same single-photon state that was used to find the mode permutation, by maximizing the cost function

$$C(\boldsymbol{\phi}) = |\left\langle \bar{n} = 1\right| W^\dagger V^\dagger F(\boldsymbol{\phi}) P_\sigma U(\boldsymbol{\theta}^*) W \left|\bar{n} = 1\right\rangle|^2 , \tag{35}$$

where the optimization is carried out only over $\boldsymbol{\phi}$, and $\boldsymbol{\theta}^*$ are fixed to the values obtained in the first step. Once complete, we have the final result $F(\boldsymbol{\phi}^*) P_\sigma U(\boldsymbol{\theta}^*) = V$ up to a global phase.

In summary, the learning process is divided into two stages. In the first stage, training is performed using bare Fock states (e.g., $|1100\rangle$), which are sufficient to learn the target unitary up to local phase shifts and mode permutations. Empirically, we find that training reliably converges to the global minimum whenever $U(\boldsymbol{\theta})$ is overparameterized. While training with multi-photon Fock states ($n > 1$) rotated by random unitaries W_i, i.e., states of the form $W_i |1100\rangle$ (as shown in 23), can in principle encode local phase information, this approach often fails in practice. The training process becomes stuck in bad local minima, a failure we attribute to the permutation symmetry of Fock states. This symmetry leads to a poor optimization landscape when optimizing over linear optical unitaries. In contrast, our three-step protocol effectively resolves this issue without increasing the required number of training states while also ensuring convergence to the correct solution. Importantly, when the number of photons scales with the number of modes ($n \propto m$), the total number of required training states becomes constant. In the optimal case where $n = m - 1$, only two training states are needed to fully reconstruct the unitary. While for the single-photon case ($n = 1$), one requires $L = m$ training states, consistent with the results presented earlier in Section 3 3.2.

We simulate our three-step protocol for learning a unitary with $m = 5$ modes, including both local phase shifts and mode permutations. The simulation is performed using different photon numbers n and varying numbers of training data L, where each training set consists of $L - 1$ Fock states with n photons and one single-photon state to learn local phases and mode permutations. Fig. S2 shows the test error C_{test} after training. As predicted, larger photon numbers require fewer training states, following the theoretical bound of $L_{\text{c}} = \text{ceil}(\frac{m-1}{n}) + 1$. To evaluate generalization performance, the test error is computed as the infidelity between the outputs of the true unitary and the trained unitary applied to a Haar-random test state $|\psi\rangle$ as

$$C_{\text{test}} = 1 - |\left\langle\psi\right| V^\dagger F(\boldsymbol{\phi}^*) P_\sigma U(\boldsymbol{\theta}^*) \left|\psi\right\rangle|^2 . \tag{36}$$

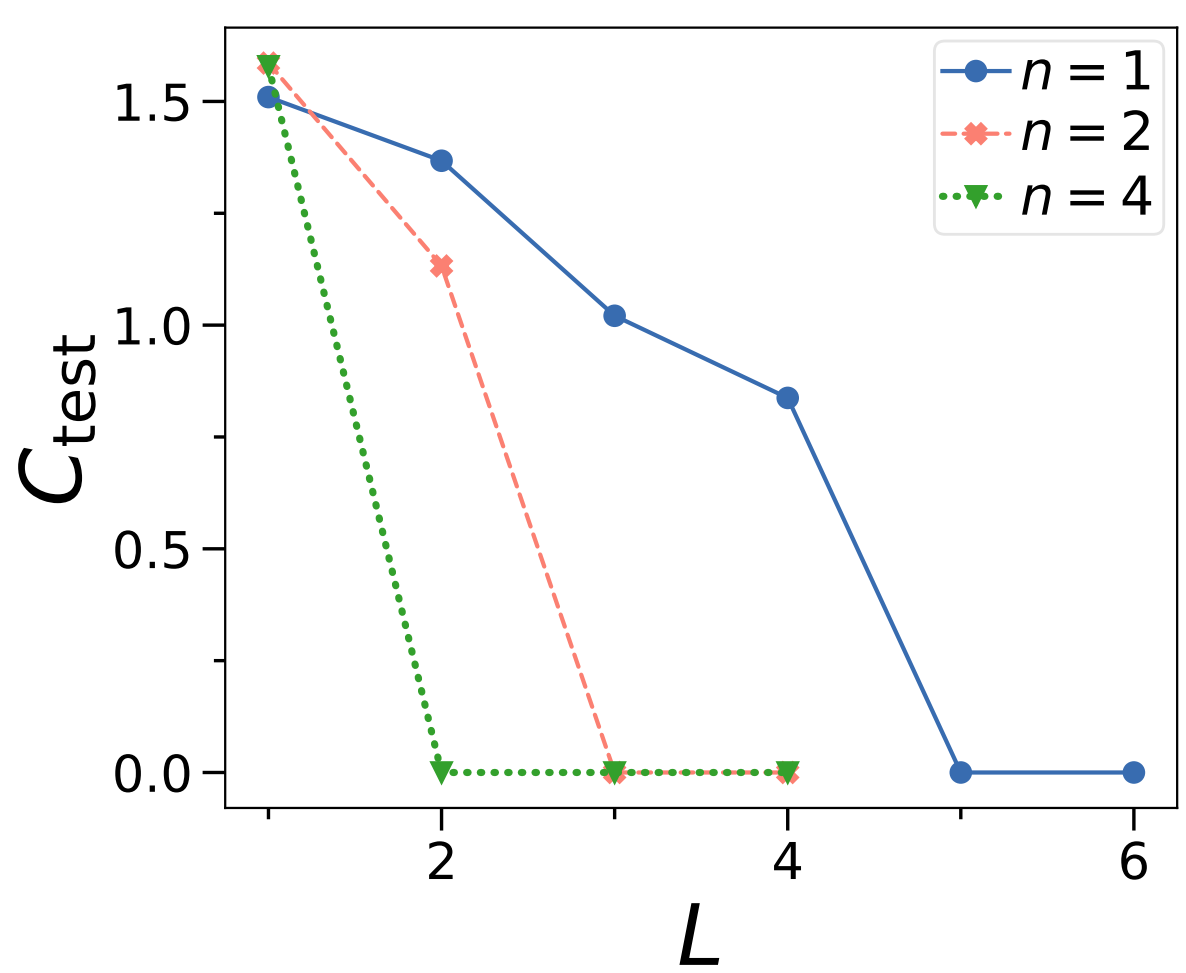

Supplementary Figure S2. **Simulation of test error for learning unitaries using the three-step protocol.** The test error is shown for different numbers of training states L and photon numbers n, for learning a target unitary with $m = 5$ modes.

4. HARDWARE EXPERIMENTS FOR LEARNING UNITARIES

4.1. Brief introduction of SPSA

To demonstrate the feasibility of photonic QML, we perform online training on our photonic quantum hardware. Starting from a random initial guess, all hardware parameters which are implemented as phase shifts in MZIs, are iteratively updated to minimize (or maximize) a given objective function.

Among various optimization strategies, gradient descent serves as the most widely-used and effective method. Gradient descent relies on gradient information, which can be obtained either by analytical expressions or finite difference approximation. Finite-difference methods are generally discouraged in quantum settings, as they demand a large number of measurements to estimate gradients with sufficient precision, making them inefficient for experimental use. In terms of analytical solutions, recent work has generalized the parameter-shift rule, originally developed for qubit-based quantum circuits, to photonic systems [12–14]. These methods allow for the exact computation of gradients, but they still scale linearly with the number of parameters. As the parameter space grows, this scaling becomes a potential computational bottleneck [15].

In this work, we adopt a pseudo gradient-based optimization method known as simultaneous perturbation stochastic approximation (SPSA) [16]. SPSA estimates the gradients with only two objective function evaluations per iteration by applying a random perturbation vector to all parameters simultaneously, regardless of the number of trainable parameters. This random vector is usually drawn from the Rademacher distribution, where each component independently takes the value ± 1 with equal probability.

Specifically, suppose we aim to minimize an objective function $f(\boldsymbol{x})$ over a parameter vector $\boldsymbol{x}$. For each iteration, we generate a random vector $\boldsymbol{\Delta}$, and evaluate $f(\boldsymbol{x}+c\boldsymbol{\Delta})$ and $f(\boldsymbol{x}-c\boldsymbol{\Delta})$ through hardware runs and measurements. The approximate gradient for $\boldsymbol{x}$ is given by

$$\delta_{\text{approx}} = \frac{f(\boldsymbol{x}+c\boldsymbol{\Delta}) - f(\boldsymbol{x}-c\boldsymbol{\Delta})}{2c\boldsymbol{\Delta}}, \tag{37}$$

where $c > 0$ is the perturbation amplitude. δ_{approx} is then used to refine the parameters $\boldsymbol{x}$.

SPSA exhibits several desirable properties for hardware-based implementations. In terms of efficiency, it avoids the linearly increasing computational cost associated with varying each parameter individually. By perturbing all parameters simultaneously, SPSA maintains a constant number of objective function evaluations per iteration, making it especially suitable for high-dimensional parameter spaces. Moreover, the stochastic nature of gradient approximation in SPSA inherently provides robustness to noise, which is a critical feature for practical applications on noisy quantum hardware. The random fluctuations introduced by SPSA mirror the stochastic noise present in hardware measurements, allowing it to operate effectively even when gradients cannot be estimated precisely. While gradient-free methods such as the Nelder–Mead algorithm have been employed to train photonic quantum neural networks[17], these methods tend to be more sensitive to hardware noise and often demonstrate inferior performance compared to SPSA [18].

In light of the above reasons, we choose the SPSA method for all physical experiments conducted in this study. To the best of our knowledge, this work represents the first application of SPSA for training photonic quantum hardware, which has only been implemented on qubit-based superconducting platforms before [19, 20].

4.2. Implementation details

As described in the main text, we test our protocol by learning a random 5×5 unitary matrix acting on five optical modes ($m = 5$), and compare the cases $n = 1$ and $n = 2$. The experiments are implemented on a six-mode programmable photonic chip, where modes $1 - 5$ are used to implement the unitary, and mode 6 is reserved for heralding when necessary.

According to the discussion in Supplementary Note 3, the first stage of learning requires at least four single-photon states ($n = 1$) and two two-photon states ($n = 2$). For $n = 1$, one photon is injected into modes 1, 2, 3, and 4 sequentially, corresponding to the Fock states $|10000\rangle, |01000\rangle, |00100\rangle$, and $|00010\rangle$. This is realized by a programmable optical switch, along with a tunable delay line to compensate for temporal variances across different optical paths. During each training epoch, each of the four states is inputted once and the output probability distributions are collected. Following the convention, we define the loss function as $\mathcal{L}(\theta) = C(\theta)$ according to 32, so that the loss value remains non-negative and converges to zero upon successful training. For $n = 2$, two identical photons are injected into mode pairs (1,2) and (3,4) in sequence, corresponding to the Fock states $|11000\rangle$ and $|00110\rangle$. We perform pseudo photon-number-resolving detection with beam splitters to measure the probability of photon bunching events where two photons reach the same output mode.

The complete experimental workflow is summarized in Algorithm 1.

Algorithm 1 Unitary learning

Input: $m \times m$ target unitary matrix V, training set containing $L-1$ n-photon Fock states $\{|\boldsymbol{s}^{(\ell)}\rangle\}_{\ell=1}^{L-1}$, loss function $\mathcal{L}$, SPSA hyperparameters c and a.
Output: Optimized unitary matrix $U(\boldsymbol{\theta}) \approx V$, $\boldsymbol{\theta} \in \mathbb{R}^q$.

Select a random seed and initialize values for $\boldsymbol{\theta}$.
repeat
    Generate a random vector $\boldsymbol{\Delta} \in \{-1, 1\}^q$ with the same dimension as $\boldsymbol{\theta}$.
    for the ℓth input Fock state $|\boldsymbol{s}^{(\ell)}\rangle$ **do**
        Set the MZI phases of the chip according to V and $\boldsymbol{\theta} + c\boldsymbol{\Delta}$.
        Perform measurement on the quantum device and get the probability vector $\boldsymbol{p}_+^{(\ell)}$.
        Set the MZI phases of the chip according to V and $\boldsymbol{\theta} - c\boldsymbol{\Delta}$.
        Perform measurement on the quantum device and get the probability vector $\boldsymbol{p}_-^{(\ell)}$.
    end for
    Calculate the approximate gradient vector $\boldsymbol{\delta} \leftarrow \dfrac{\sum_\ell \mathcal{L}(\boldsymbol{p}_+^{(\ell)}) - \sum_\ell \mathcal{L}(\boldsymbol{p}_-^{(\ell)})}{2c\boldsymbol{\Delta}}$.
    Update the trainable parameters $\boldsymbol{\theta} \leftarrow \boldsymbol{\theta} - a\boldsymbol{\delta}$.
until convergence

Regarding SPSA hyperparameters, the perturbation and learning rate are initially set as $c = 0.3$ and $a = 4$, and exponentially decay with the epoch number. We adopt decay coefficients recommended in prior work[19–21], with $\alpha = 0.602$ and $\gamma = 0.101$. These hyperparameters show ideal convergence performance in numerical simulations for both $n = 1$ and $n = 2$ cases, and are further applied in the hardware experiments.

The result of the first training round has been presented in Fig. 3b in the main text, where the target unitary V is learned up to local phases and mode permutations. Here, we complete the learning process and implement the remaining steps as described in Supplementary Note 3. Specifically, a single-photon state is prepared to identify the permutation P_σ and learn the local phase unitary $F(\phi)$. To monitor the learning process, we compute the matrix closeness as $1 - \left|\frac{1}{m}\mathrm{tr}(V^\dagger F(\boldsymbol{\phi}) P_\sigma U(\boldsymbol{\theta}^*))\right|$, consistent with the definition used in the main text. It quantifies the overlap between the learned and target unitaries up to an unobservable global phase. A perfect learning result indicates that $V^\dagger F(\boldsymbol{\phi}) P_\sigma U(\boldsymbol{\theta}^*)$ is an identity matrix up to some global phase, so the matrix closeness should be close to zero.

The experimental results of second-round learning are shown in Fig. S3. Both $n = 1$ and $n = 2$ cases exhibit rapid convergence in approximately 150 training epochs, and the final similarity values are very close to one within the noise limits of the hardware. These results provide experimental support for the feasibility and robustness of our theoretical three-step learning protocol.

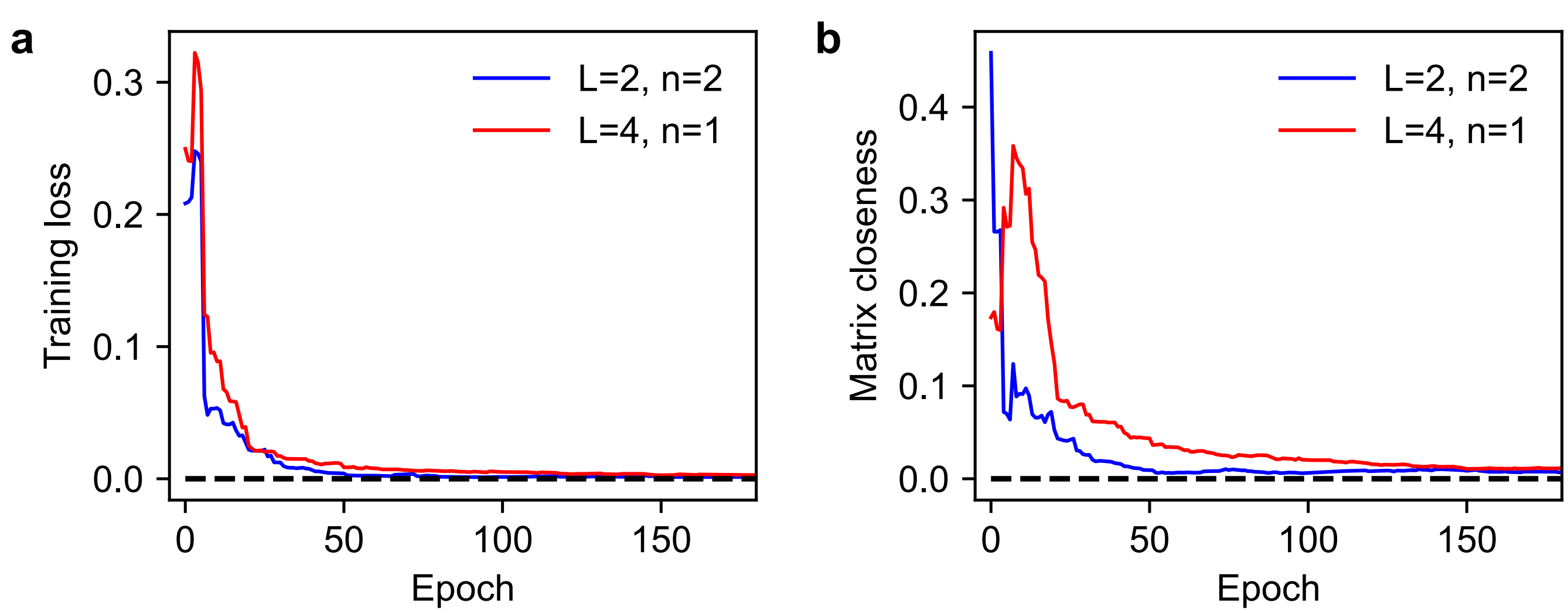

Supplementary Figure S3. **Learning local phases of a unitary in the second round.** (a) Training loss and (b) matrix closeness for photon numbers $n = 1$ (red curves) and $n = 2$ (blue curves). In both cases, local phases are successfully learned, starting from the trained unitaries obtained in the first round of learning.

5. QUANTUM METRIC LEARNING WITH MULTI-PHOTON STATES

5.1. Theoretical framework

The goal of metric learning is to learn a representation in which similar data points are mapped closer together, while dissimilar ones are mapped farther apart. For an in-depth overview of metric learning techniques, we refer interested readers to comprehensive survey papers such as [22].

Here, we show that the mathematical structure of linear optical circuits is naturally compatible with metric learning. In many representative studies of metric learning [23–25], a neural network is trained to map each input to a normalized feature vector, such that all outputs lie on the surface of a unit hypersphere. This geometric constraint facilitates angular-based loss functions, which are widely used to enhance class separability. In our quantum setting, a variational linear optical circuit ansatz encodes each data point onto its parameters through some mapping scheme, and produces output probability distributions over Fock basis states, denoted by $\{p_i\}$ and $\{q_i\}$ for two respective data points. Since these are valid probability distributions $\sum_i p_i = \sum_i q_i = 1$, their square roots $\{\sqrt{p_i}\}$ and $\{\sqrt{q_i}\}$ naturally form unit vectors in a high-dimensional vector space.

A widely-used similarity measure for probability distributions is the Bhattacharyya coefficient $S_{\mathrm{C}}(p,q) = \sum_i \sqrt{p_i q_i}$, which is mathematically equivalent to the cosine similarity between $\{\sqrt{p_i}\}$ and $\{\sqrt{q_i}\}$. This enables us to interpret the output probability vectors of the photonic circuit as hyperspherical feature embeddings, analogous to those used in classical deep metric learning. We therefore define a loss function that encourages small angular distances (i.e., high cosine similarity) between embeddings of similar inputs, and large angular distances between dissimilar inputs.

A key hyperparameter in metric learning is the margin, which defines the minimum required separation between positive and negative pairs in the embedding space. The choice of margin significantly affects the difficulty of the learning task and the overall performance of the model. A larger margin imposes a stricter constraint by requiring that negative pairs be more widely separated, which makes optimization more challenging. However, this encourages the model to learn more discriminative features, thereby improving its ability to generalize and distinguish between different classes.

In this study, we integrate core concepts of metric learning into the framework of linear quantum optics. We introduce a hyperparameter t, referred to as the angular distance margin, to investigate different learning difficulty scenarios. A loss function $\mathcal{L}$ regularizes the training process as follows: for **positive** pairs (data points with the same label), the cosine similarity between the output distributions should be larger than t, while for **negative** pairs (data points with different labels), the cosine similarity should be smaller than t. Specifically, $\mathcal{L}$ is defined as

$$\mathcal{L} = \frac{1}{N_{\mathrm{total}}}\left[\sum_{\substack{(m,n)\\ y^{(m)}=y^{(n)}}} \mathrm{ReLU}\left(t - \sum_i \sqrt{p_i^{(m)} p_i^{(n)}}\right) + \sum_{\substack{(m,n)\\ y^{(m)}\neq y^{(n)}}} \mathrm{ReLU}\left(\sum_i \sqrt{p_i^{(m)} p_i^{(n)}} - t\right)\right], \tag{38}$$

where $\mathrm{ReLU}(\cdot) = \max(0,\cdot)$ is the standard rectified linear unit activation function, N_{total} is the number of all sample pairs within a training batch, $y^{(n)}$ is the class label of the nth data point, and $p^{(n)}$ is the measured real-valued probability vector for the nth data point, respectively. We fix $t = 0.9$ throughout all simulations and physical experiments presented in the main text.

5.2. Experimental implementation details

The vowel dataset in our work has been employed in multiple studies to benchmark novel machine learning hardware platforms [26–28]. The dataset consists of 12-dimensional data vectors of formant frequencies extracted from audio recordings, categorized into seven vowel classes: 'ae', 'ah', 'aw', 'er', 'ih', 'iy' and 'uw'. Each class contains 37 samples. We randomly split the dataset into training and test sets by a 70/30 ratio, while maintaining class balance across both sets. As a result, the training and test sets contain $26 \times 7 = 182$ and $11 \times 7 = 77$ data points, respectively.

The schematic diagram of the variational circuit used for learning is shown in Fig. S4. To maximize the number of variational parameters on our square mesh of MZIs [3], we adopt the trainable linear scaling scheme proposed in [27] for data encoding. Specifically, we first perform min-max normalization across the whole dataset, so that all input components x_{1-12} lie in $[0,1]$. For each component x_i, we assign two trainable parameters k_i and b_i, and compute the value of $\hat{x}_i = k_i x_i + b_i$, which is then mapped to the corresponding phase shifter in the circuit. The remaining MZIs carry free trainable parameters $(\varphi_{1-4}, \theta_{1-4})$. Because the external phases of the front MZIs do not affect the measured probabilities, we fix them to zero, and only the internal phases (θ_{5-8}) are trainable.

Similar to the unitary learning task, we employ SPSA method for online training. At each training step, we randomly sample five instances per class, resulting in $5 \times 7 = 35$ data points per mini-batch. The training loss is

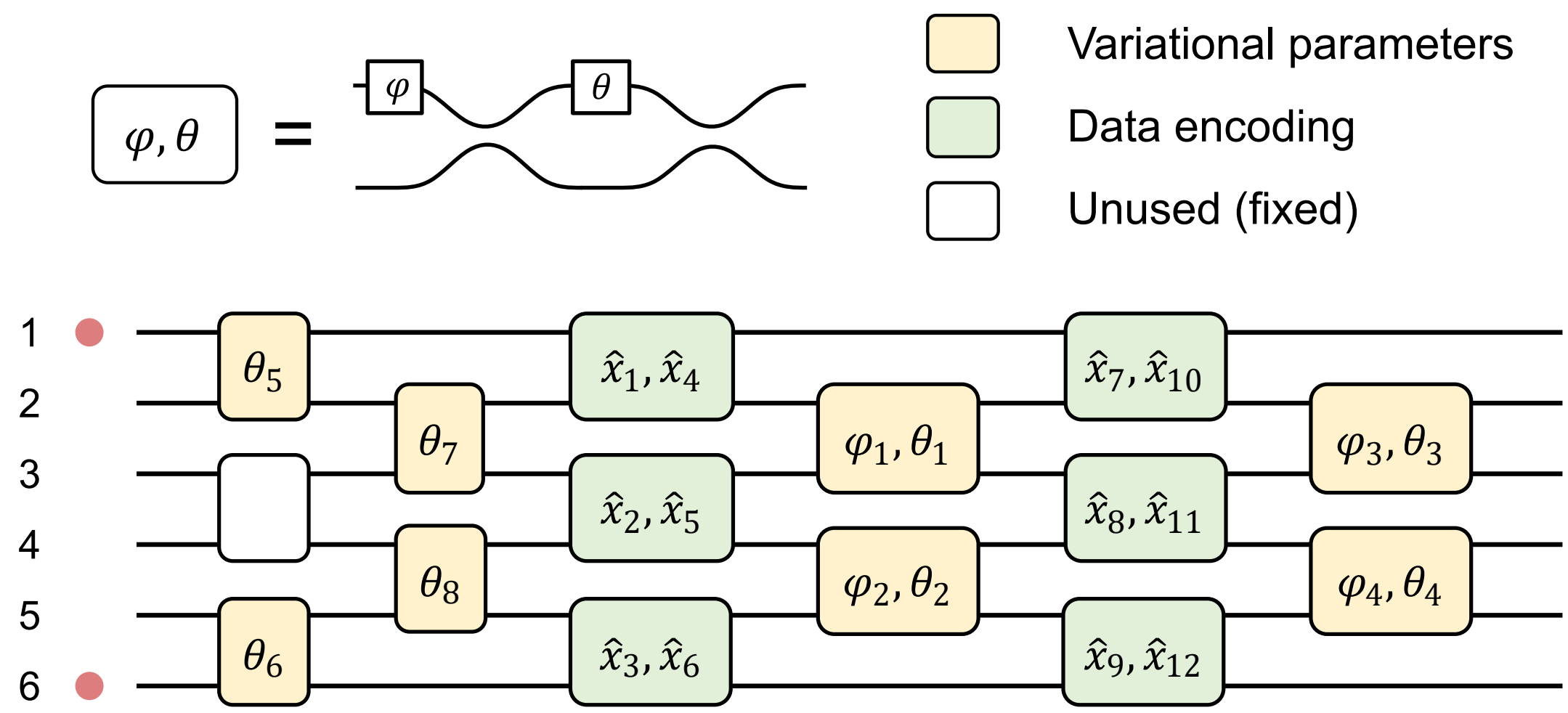

Supplementary Figure S4. **Schematic diagram of the variational circuit used for quantum metric learning.** The phase shifters of the six-port square mesh of MZIs are configured either to encode input data components or to carry trainable parameters used for optimization.

calculated and optimized over all pairwise combinations in the mini-batch. The experimental pipeline is shown in Algorithm 2.

Algorithm 2 Quantum metric learning

Input: Training and test set of data points $\{\boldsymbol{x}\}$ consisting of 12-dimensional real-valued vectors, class labels $\{y\}$, loss function $\mathcal{L}$, SPSA hyperparameters c and a, batch size N.
Output: Optimized parameters $\boldsymbol{\lambda} \in \mathbb{R}^q$, including $\varphi_{1-4}, \theta_{1-8}, k_{1-12}, b_{1-12}$ as defined above.

```
Select a random seed and initialize values for λ.
repeat
    // Training stage
    Randomly select a mini-batch of N data points from the training set.
    Generate a random vector Δ ∈ {−1, 1}^q with the same dimension as λ.
    for the nth data point x^(n) do
        Set the corresponding MZI phases of the chip according to x^(n) and λ + cΔ.
        Perform measurement on the quantum device and get the probability vector p_+^(n).
        Set the corresponding MZI phases of the chip according to x^(n) and λ − cΔ.
        Perform measurement on the quantum device and get the probability vector p_−^(n).
    end for
    Calculate the approximate gradient vectors δ_i ← (Σ_n L(p_+^(n), y^(n)) − Σ_n L(p_−^(n), y^(n))) / 2cΔ.
    Update the trainable parameters λ ← λ − aδ.
    // Evaluation stage
    for the nth data point x^(n) in the test set do
        Set the corresponding MZI phases of the chip according to x^(n) and λ.
        Perform measurement on the quantum device and get the probability vector p^(n).
    end for
    Calculate the average loss value on the test set.
until convergence
```

Regarding the hardware setup, for the $n = 2$ case, two identical photons are generated by a spontaneous parametric down-conversion (SPDC) source and injected into ports 1 and 6. For the $n = 1$ case, only one of the two SPDC photons passes through a beam splitter whose outputs are coupled to ports 1 and 6, and the other photon is used for heralding. This configuration guarantees that the same optical paths and phase encoding structure are involved in both settings, enabling a fair comparison.

The experimental results are shown in Fig. 4c and 4d in the main text. Here, we further visualize the Gram matrix on the test set for $n = 2$ with a diverging colour map (Fig. S5). The matrix exhibits a clear 7×7 block structure, corresponding to the seven classes of the dataset. Although the angular distance margin was set to be $t = 0.9$ during training, we observe that most inter-class distances (off-diagonal blocks) fall well below 0.9. This suggests that the

model has successfully learned a distance measure that clusters positive pairs closely and separates negative pairs, independently of the specific numerical value of t.

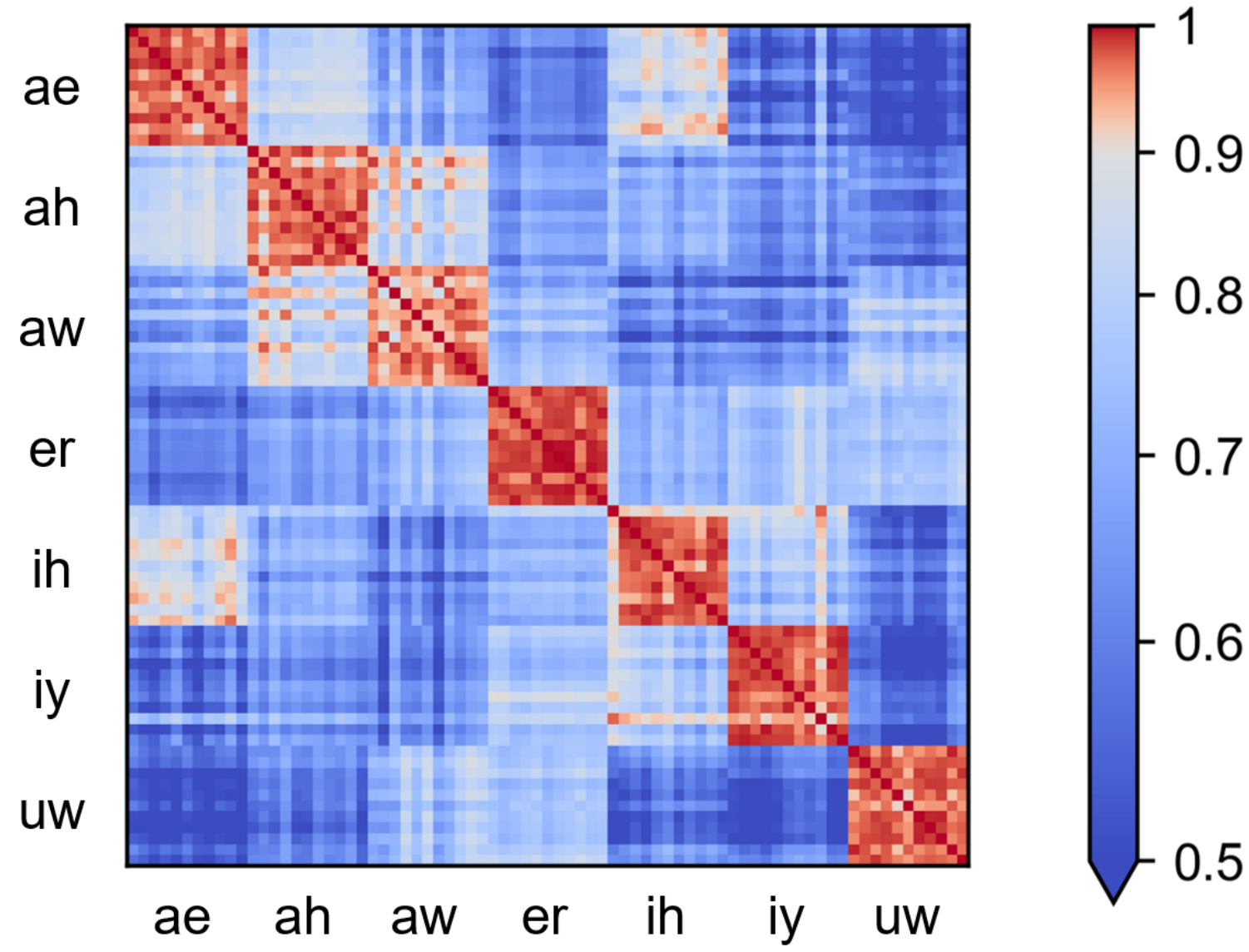

Supplementary Figure S5. **Experimental Gram matrix on the test set for photon number $n = 2$ and margin $t = 0.9$.** This result corresponds to Fig. 4d in the main text.

To further demonstrate the robustness of online training on our photonic quantum hardware, we performed additional experiments with a different margin value $t = 0.95$, as shown in Fig. S6. This value approaches the average fidelity between theoretical and experimentally measured probability distributions of our photonic chip (around 0.98), making the training task more sensitive to noise and hence more challenging. Despite this, similar results are obtained as in the case of $t = 0.9$, where only a slight confusion between the classes 'ah' and 'aw' remains. This is consistent with the results of previous research [27, 28], as the instances of these two classes are intrinsically similar.

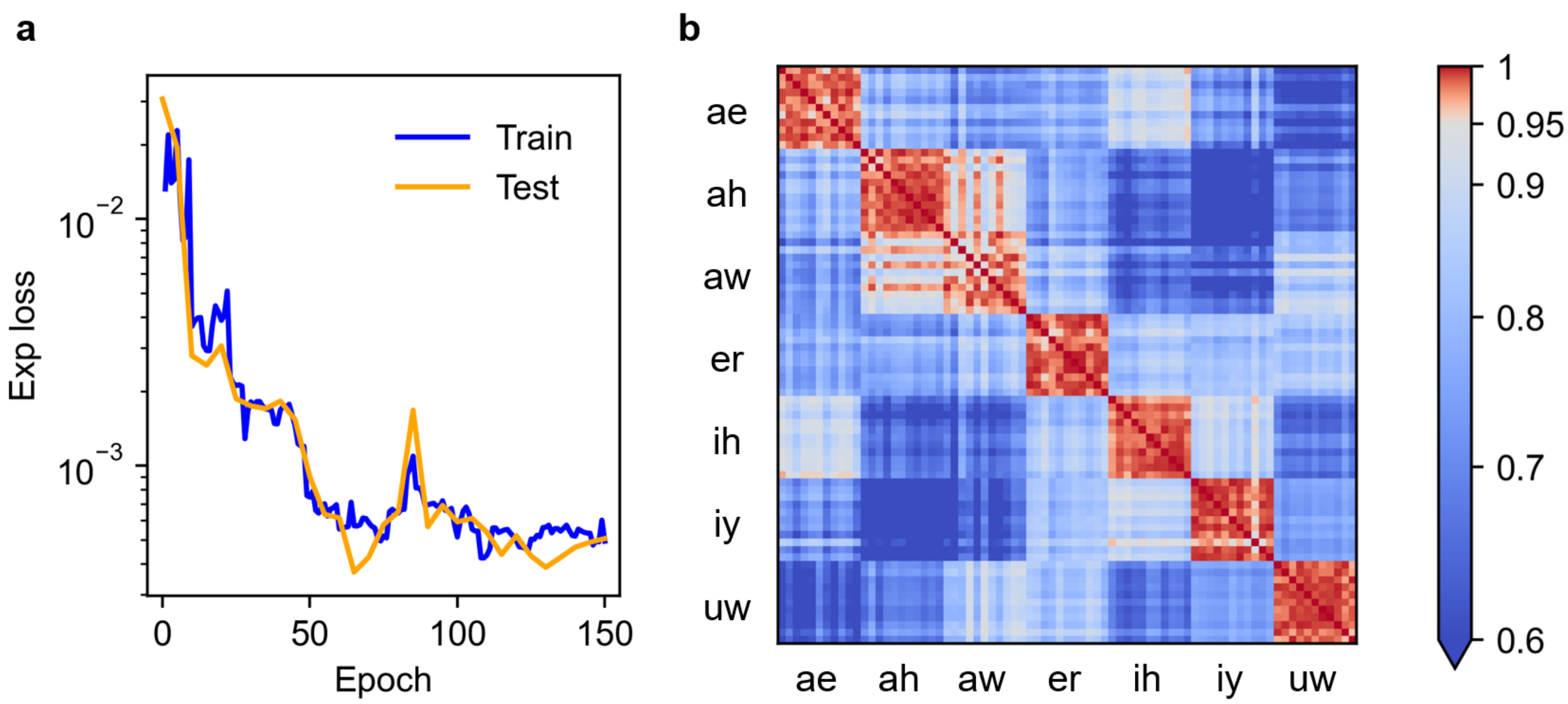

Supplementary Figure S6. **Experimental result of quantum metric learning for photon number $n = 2$ and margin $t = 0.95$.** a) Experimental training and test loss over epochs. b) Gram matrix on the test set after training, which reveals inter-class and intra-class similarities.

5.3. Numerical simulations with larger photon numbers

Metric learning provides a good opportunity for comparing the model capability and trainability of different photonic configurations. In conventional classification tasks, fully-connected layers are often required to map model outputs to discrete class labels. However, since different combinations of photon and mode numbers yield output probability vectors of varying dimensions, fully-connected layers introduce varying numbers of trainable parameters, which complicates fair comparisons across models. In contrast, metric learning focuses on the inner products between probability vectors rather than the vectors themselves, thereby circumventing the issue of varying output dimensions arising from different photon and mode numbers.

In the main text (Fig. 4b), we display the performance scaling from photon number $n = 1$ to $n = 5$ under the margin hyperparameter $t = 0.9$. Here, we extend the analysis by training models with alternative values of $t = 0.5, 0.6, 0.7, 0.8$ while keeping all other settings identical. The final loss values on the test set are shown in Fig. S7. As the value of t decreases, the model is required to enforce larger angular distances between negative pairs, making the learning task progressively more difficult. This is reflected in the increasing loss values from $t = 0.9$ to $t = 0.5$. At the same time, we observe that the marginal improvement in loss diminishes with increasing photon number, a trend that holds across different values of t. This behavior is consistent with the sublinear scaling of the QFIM rank with respect to the photon number n. The only exceptions occur for $n = 5$ at $t = 0.5$ and $t = 0.6$. This may be attributed to the fact that, for larger photon numbers, the trainable parameters become more highly correlated in the output probabilities, and the gradient landscape becomes more complex due to the increased difficulty of computing matrix permanents, which affects the optimization performance.

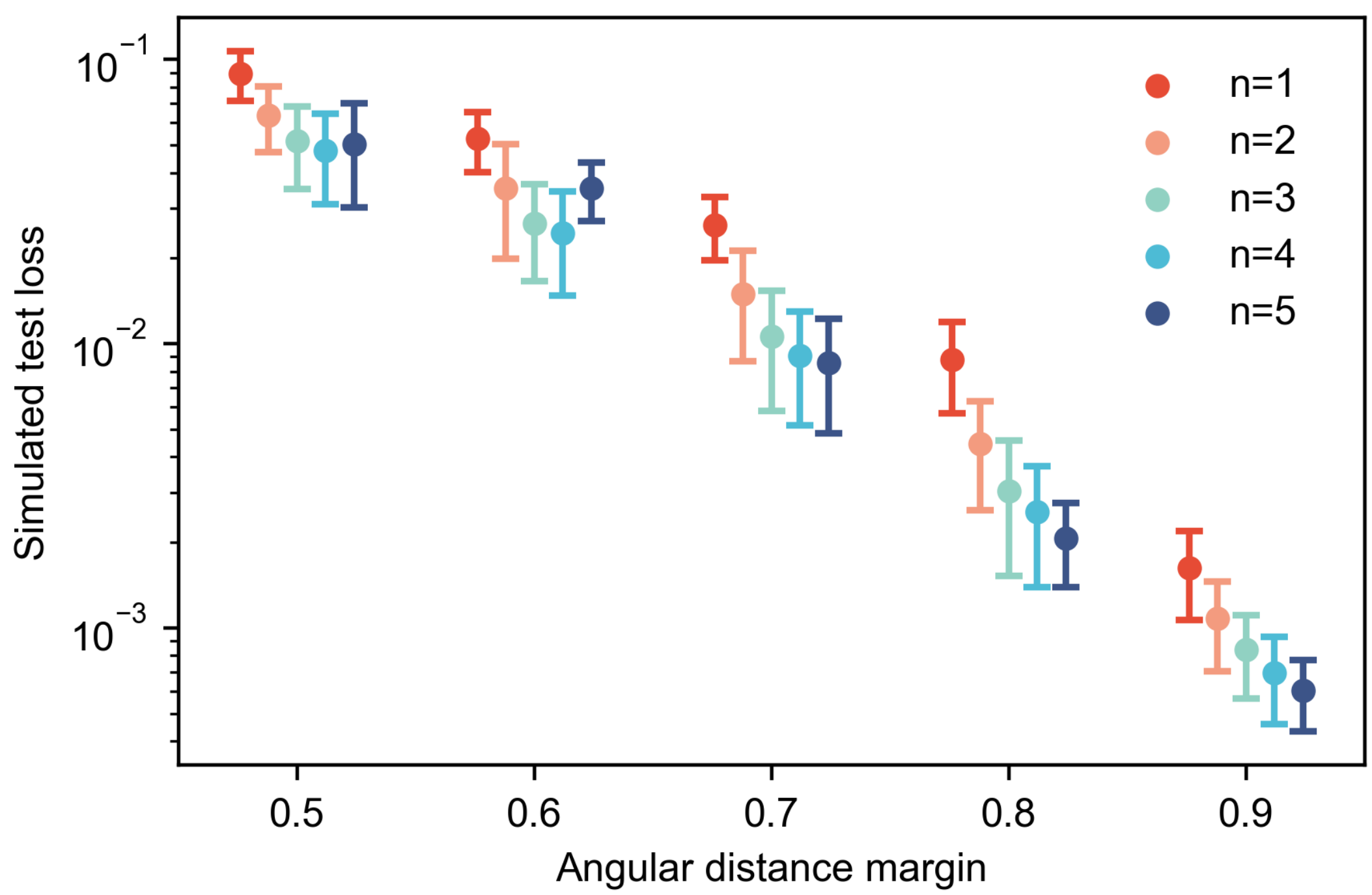

Supplementary Figure S7. **Simulated test loss for different photon numbers** $n = 1, 2, 3, 4, 5$ **under varying hyperparameter values** $t = 0.5, 0.6, 0.7, 0.8, 0.9$**.** The results illustrate how decreasing t increases the learning difficulty and highlight the diminishing performance gain with larger n.

SUPPLEMENTARY REFERENCES

[1] Aaronson, S. & Arkhipov, A. The Computational Complexity of Linear Optics. *Proceedings of the 43rd annual ACM symposium on Theory of computing - STOC '11* **9**, 333 (2010-11-14). 1011.3245.
[2] Reck, M., Zeilinger, A., Bernstein, H. J. & Bertani, P. Experimental realization of any discrete unitary operator. *Physical Review Letters* **73**, 58–61 (1994).
[3] Clements, W. R., Humphreys, P. C., Metcalf, B. J., Kolthammer, W. S. & Walmsley, I. A. Optimal design for universal multiport interferometers. *Optica* **3**, 1460–1465 (2016).
[4] Haug, T., Bharti, K. & Kim, M. Capacity and quantum geometry of parametrized quantum circuits. *PRX Quantum* **2**, 040309 (2021).
[5] Liu, J., Yuan, H., Lu, X.-M. & Wang, X. Quantum fisher information matrix and multiparameter estimation. *Journal of Physics A: Mathematical and Theoretical* **53**, 023001 (2020).
[6] Haug, T. & Kim, M. Generalization of quantum machine learning models using quantum fisher information metric. *Physical Review Letters* **133**, 050603 (2024).
[7] Polcari, J. Representing unitary matrices by independent parameters. Tech. Rep., Working Paper, Rev 0, October, 2016 (2016).
[8] Brask, J. B. Gaussian states and operations–a quick reference. *arXiv preprint arXiv:2102.05748* (2021).
[9] Larocca, M., Ju, N., García-Martín, D., Coles, P. J. & Cerezo, M. Theory of overparametrization in quantum neural networks. *Nature Computational Science* **3**, 542–551 (2023).
[10] You, X., Chakrabarti, S. & Wu, X. A convergence theory for over-parameterized variational quantum eigensolvers. *arXiv preprint arXiv:2205.12481* (2022).
[11] Vlasov, A. Y. Permanents, bosons and linear optics. *Laser Physics Letters* **14**, 103001 (2017).
[12] Pappalardo, A. *et al.* A photonic parameter-shift rule: Enabling gradient computation for photonic quantum computers. *arXiv preprint arXiv:2410.02726* (2024).
[13] Facelli, G. *et al.* Exact gradients for linear optics with single photons. *arXiv preprint arXiv:2409.16369* (2024).
[14] Cimini, V. *et al.* Variational quantum algorithm for experimental photonic multiparameter estimation. *npj Quantum Information* **10**, 26 (2024).
[15] Gacon, J., Zoufal, C., Carleo, G. & Woerner, S. Simultaneous perturbation stochastic approximation of the quantum fisher information. *Quantum* **5**, 567 (2021).
[16] Spall, J. C. Multivariate stochastic approximation using a simultaneous perturbation gradient approximation. *IEEE transactions on automatic control* **37**, 332–341 (1992).
[17] Maring, N. *et al.* A versatile single-photon-based quantum computing platform. *Nature Photonics* **18**, 603–609 (2024).
[18] Pellow-Jarman, A., Sinayskiy, I., Pillay, A. & Petruccione, F. A comparison of various classical optimizers for a variational quantum linear solver. *Quantum Information Processing* **20**, 202 (2021).
[19] Havlíček, V. *et al.* Supervised learning with quantum-enhanced feature spaces. *Nature* **567**, 209–212 (2019).
[20] Glick, J. R. *et al.* Covariant quantum kernels for data with group structure. *Nature Physics* **20**, 479–483 (2024).
[21] Spall, J. C. Implementation of the simultaneous perturbation algorithm for stochastic optimization. *IEEE Transactions on aerospace and electronic systems* **34**, 817–823 (1998).
[22] Kaya, M. & Bilge, H. Ş. Deep metric learning: A survey. *Symmetry* **11**, 1066 (2019).
[23] Liu, W. *et al.* Sphereface: Deep hypersphere embedding for face recognition. In *Proceedings of the IEEE conference on computer vision and pattern recognition*, 212–220 (2017).
[24] Wang, H. *et al.* Cosface: Large margin cosine loss for deep face recognition. In *Proceedings of the IEEE conference on computer vision and pattern recognition*, 5265–5274 (2018).
[25] Deng, J., Guo, J., Xue, N. & Zafeiriou, S. Arcface: Additive angular margin loss for deep face recognition. In *Proceedings of the IEEE/CVF conference on computer vision and pattern recognition*, 4690–4699 (2019).
[26] Romera, M. *et al.* Vowel recognition with four coupled spin-torque nano-oscillators. *Nature* **563**, 230–234 (2018).
[27] Wright, L. G. *et al.* Deep physical neural networks trained with backpropagation. *Nature* **601**, 549–555 (2022).
[28] Momeni, A., Rahmani, B., Malléjac, M., Del Hougne, P. & Fleury, R. Backpropagation-free training of deep physical neural networks. *Science* **382**, 1297–1303 (2023).
